# Reimagining Spinal Surgery at the Nanoscale: Smart Implants, Targeted Therapies, and Translational Challenges

**DOI:** 10.3390/biology15161400

**Published:** 2026-08-15

**Authors:** Alexander Shao-Rong Pang, Kimberley Yun-Lin Pang, Zi Qiang Glen Liau, Arun-Kumar Kaliya-Perumal, Jacob Yoong-Leong Oh, Dinesh Kumar Srinivasan

**Affiliations:** 1 Yong Loo Lin School of Medicine, National University of Singapore, Singapore 117597, Singapore; alexandersrp@u.nus.edu; 2 Department of Orthopaedic Surgery, Tan Tock Seng Hospital, Singapore 308433, Singapore; 3 Department of Orthopaedic Surgery, National University Hospital, Singapore 119074, Singapore; 4 Rehabilitation Research Institute of Singapore, Nanyang Technological University, Singapore 308232, Singapore; arunkumar.kp@ntu.edu.sg; 5 Department of Anatomy, Yong Loo Lin School of Medicine, National University of Singapore, Singapore 117594, Singapore

**Keywords:** nanotechnology, nanostructured implant surfaces, osseointegration, nano-drug delivery systems, intervertebral disc degeneration

## Abstract

Spinal pathologies, including degenerative disc disease, spinal cord injury, and conditions requiring spinal fusion, pose a substantial global health burden. While contemporary interventions provide symptomatic relief, achieving durable tissue repair in biologically compromised environments remains a critical challenge. This narrative review synthesizes the current literature on three major nanotechnology applications in spine care: nanostructured implant surfaces, nanoparticle-enhanced bone grafts, and nano-drug delivery systems (NDDSs). Preclinical evidence indicates that nanoscale surface modifications and nanoparticle-augmented synthetic grafts significantly enhance osseointegration and bone fusion by mimicking the native extracellular matrix. Furthermore, in animal models of intervertebral disc degeneration, NDDSs utilizing polymeric nanoparticles and exosomes facilitate sustained, stimuli-responsive therapeutic delivery into the avascular disc space. Although early clinical data on nanostructured cages demonstrate reduced subsidence and stable long-term fusion, the direct translation of these robust preclinical outcomes to widespread clinical efficacy faces substantial hurdles. Significant translational barriers include stringent Class III regulatory classifications, sparse long-term safety data regarding nanoparticle biodistribution, and scale-up manufacturing challenges such as batch variability. Future progress relies on artificial intelligence-guided design, three-dimensional (3D) bioprinting, and multifunctional “smart” nanomaterials. Ultimately, close collaboration among materials scientists, clinicians, and regulatory bodies is essential to safely bridge the gap between preclinical innovation and predictable clinical therapeutic success.

## Simple Summary

Millions of people worldwide suffer from spinal problem such as degenerative disc disease, spinal cord injuries, and conditions that require bones in the spine to be fused together. Current treatments can reduce pain and improve function, but often fail to achieve long-lasting healing, especially in patients with weakened bones or complex medical conditions. Nanotechnology, the science of manipulating materials at extremely small scales, about one-thousandth the width of a human hair, offers promising new solutions. By engineering implant surfaces and bone grafts at this scale, researchers can mimic the natural structure of bone, helping the body integrate implants more effectively, promoting stronger bone fusion. Additionally, nano-sized drug carriers can deliver medications directly to damaged spinal discs, overcoming challenges like poor blood supply in these areas. Early studies in animals, and some human trials show encouraging results, including reduced implant sinking and improved healing. However, significant obstacles remain before these technologies become readily available, including strict safety regulations, limited long-term data, and manufacturing difficulties. Future advances will require collaboration between scientists, doctors, and regulatory agencies to ensure these innovative treatments are safe, effective, and accessible to patients who need them most.

## 1. Introduction

Spinal pathologies, including degenerative disc disease, spinal cord injury, and conditions requiring spinal fusion, represent a significant global health burden affecting millions of patients worldwide [1,2,3]. Current treatment strategies, ranging from conservative management to surgical intervention, have substantially improved pain relief, neurological recovery, and functional outcomes for many patients [4,5]. Nevertheless, challenges remain particularly in achieving durable tissue repair in osteoporosis or revision surgeries, modulating the biological environment of degeneration or injury, and improving long-term regenerative outcomes [6]. In this context, the emergence of nanotechnology has introduced a paradigm shift in spinal surgery, offering innovative solutions that operate at the cellular and molecular levels to enhance therapeutic outcomes and potentially solve the aforementioned unmet needs for spinal fusion [7,8].

Nanotechnology encompasses the manipulation of materials at the nanoscale [typically 1–100 nanometers (nm)], enabling unique physical, chemical, and biological properties that differ fundamentally from their bulk counterparts. In the context of spinal surgery, nanotechnology applications can broadly be categorised into structural and therapeutic approaches. Structural applications include nanoscale surface modification of spinal implants to enhance osseointegration and support tissue regeneration. Therapeutic applications include nano-drug delivery systems (NDDSs) for targeted therapeutic interventions such as nanostructured scaffolds for tissue regeneration, and nanoparticle-mediated approaches for spinal cord repair [7,8,9]. These nanoscale interventions can precisely modulate cellular interactions, control drug release kinetics, and create biomimetic microenvironments that promote healing and regeneration.

The integration of both structural and therapeutic nanotechnology into spinal surgery may help address specific biological challenges of conventional treatments. Nanostructured surfaces on spinal implants can significantly improve bone-implant integration by mimicking the natural extracellular matrix (ECM) architecture, thereby enhancing mesenchymal stem cell (MSC) recruitment, osteoblast differentiation, and ultimately bone formation at the implant interface [10,11]. NDDSs overcome the challenges of poor bioavailability and short therapeutic half-lives by providing sustained, targeted release of bioactive molecules, such as small-molecule drugs, growth factors, genes, and RNA molecules, directly at the site of pathology [9,12]. Furthermore, nanomaterials demonstrate exceptional biocompatibility, biodegradability, and the capacity to cross cellular barriers, making them ideal candidates for treating complex spinal conditions [13].

This narrative review provides a comprehensive examination of potential nanotechnology applications in spinal surgery, with particular emphasis on recent advances and future directions. We synthesize current knowledge regarding nanoparticle-enhanced spinal fusion strategies, nanostructured implant surfaces, and NDDSs for intervertebral disc degeneration (IVDD) from both human and animal studies, with animal models serving as a key bridge for bone tissue engineering and translational disease modelling [14]. There were no systematic search restrictions. Literature was selected to represent key mechanistic insights, recent advances, and translational progress across structural and therapeutic domains, prioritizing studies that illustrate fundamental principles and clinical relevance. By consolidating mechanistic insights with practical design principles, we aim to chart a path from fundamental nanoscale interactions to clinically impactful therapeutic interventions that can transform outcomes for patients with spinal pathologies.

## 2. Nanotechnology Applications in Spinal Surgery

### 2.1. Nanoparticle-Enhanced Spinal Fusion

The mechanisms by which nanoparticles promote bone regeneration in spinal fusion have become increasingly well-defined, revealing multiple complementary pathways. Nanoparticles steer stem cell fate through mechanotransduction and signalling pathways including wingless-related integration site/beta-catenin (Wnt/β-catenin), bone morphogenetic protein (BMP)/Smad (Sma- and Mad-related protein), and mitogen-activated protein kinase (MAPK), while also modulating controlled ion release, redox balance, protein-corona dynamics, and macrophage polarization toward pro-healing phenotypes [15].

Spinal degeneration is highly prevalent, but effective regenerative strategies remain limited; consequently, spinal fusion has been the surgical treatment of choice [16,17]. Fusion aims to achieve solid bony union between adjacent vertebrae; however, in patients with osteoporosis or other comorbidities, fusion rates remain suboptimal [18,19,20,21,22]. Nanoparticle-based strategies have demonstrated significant potential for enhancing fusion outcomes through multiple mechanisms in animal models. In a rat model, lactoferrin-anchored tannylated mesoporous silica nanomaterials were shown to promote bone fusion by serving as osteogenic growth factors while simultaneously providing antiviral, anti-inflammatory, and antibacterial effects. In this model, these nanomaterials were seen to induce radiological fusion, stimulate new bone formation, increase osteocalcin expression, and promote angiogenesis at the fusion site [23]. These findings collectively demonstrate that nanoparticles enhance fusion through complementary mechanisms, such as osteogenic differentiation, angiogenesis, and immunomodulation, rather than a single pathway. This suggests that multifunctional nanomaterial design may optimize clinical outcomes.

In a rat spinal fusion model, poly(lactic-co-glycolic acid)/beta-tricalcium phosphate (PLGA/β-TCP) composite scaffolds incorporating salvianolic acid B promoted bone fusion through enhanced angiogenesis and osteogenesis, with dose-dependent increases in new bone formation, mineral apposition rate, and vessel density within the scaffold [24]. In another rat spinal fusion model conducted by Notani et al., exfoliated carbon nanofibers combined with BMP-2 enhanced fusion rates (45.5% at 8 weeks vs. 0% for control), demonstrating synergistic effects of nanomaterials with osteogenic growth factors [25]. In a rabbit posterolateral lumbar spinal fusion model, a nanostructured magnesium-doped hydroxyapatite/type I collagen scaffold augmented with platelet-rich plasma achieved 75% fusion rate (vs. 12.5% with scaffold alone), with significant upregulation of osteogenic genes including runt-related transcription factor 2 (*RUNX2*) (8–20-fold), secreted protein acidic and rich in cysteine (*SPARC*) (19–55-fold), and secreted phosphoprotein 1 (*SPP1*) (46–91-fold) [26]. The consistent upregulation of master osteogenic transcription factors across multiple animal models establishes that nanoparticle-mediated fusion enhancement operates through direct genetic reprogramming of bone-forming cells, providing mechanistic rationale for clinical translation.

Nanosynthetic silicated calcium phosphate putties used as autograft extenders achieved 87.5% bilateral fusion rates at 12 weeks and 100% fusion at 26 weeks in rabbit posterolateral fusion models, comparable to or exceeding autograft alone. Histology demonstrated new bone forming directly on nanoparticle surfaces within the fusion mass, with consistent bridging between transverse processes [27]. These findings suggest that properly designed synthetic nanomaterials can reduce or eliminate the need for extensive autograft harvest, thereby avoiding donor site morbidity while maintaining fusion efficacy.

Nano-hydroxyapatite (Nano-HA) coatings on biphasic calcium phosphate bone grafts significantly improve fusion rates, new bone formation, and biomechanical properties of the fusion mass in rabbit posterolateral fusion. The nano-HA coating enhances bioactivity, biocompatibility, and degradability of the artificial bone graft, while combined magnetic field treatment further augments these effects by increasing BMP-2 and transforming growth factor beta 1 (TGF-β1) expression in the fusion region [28]. These findings suggest that combination strategies incorporating nanomaterials with other therapeutic modalities may achieve superior fusion outcomes compared to either approach alone. This hierarchical biomimicry principle combining nano- and micro-scale features, has guided the design of next-generation implant surfaces that recapitulate the natural bone microarchitecture more faithfully than single-scale modifications. Further clinical studies will be required to determine their safety, effectiveness, and translational feasibility in human spinal fusion. The outcomes of these preclinical studies are tabulated below (Table 1).

The clinical translation of nanoparticle-based bone regeneration strategies has begun, with several systems reaching clinical application including Signafuse (bioactive calcium phosphate composite), nanoLOCK (nanostructured titanium spinal implant), and Vitoss (synthetic bone graft of nanocrystalline calcium phosphate) [29,30,31,32]. However, gaps in long-term safety data, Good Manufacturing Practices (GMP), and regulatory frameworks continue to limit broader adoption [15]. The complexity of nanoparticle characterization, batch-to-batch variability, and challenges in scaling up production from laboratory to commercial quantities represent significant barriers to commercialization [33].

### 2.2. Nanostructured Implant Surfaces for Enhanced Osseointegration

**Figure 1:**
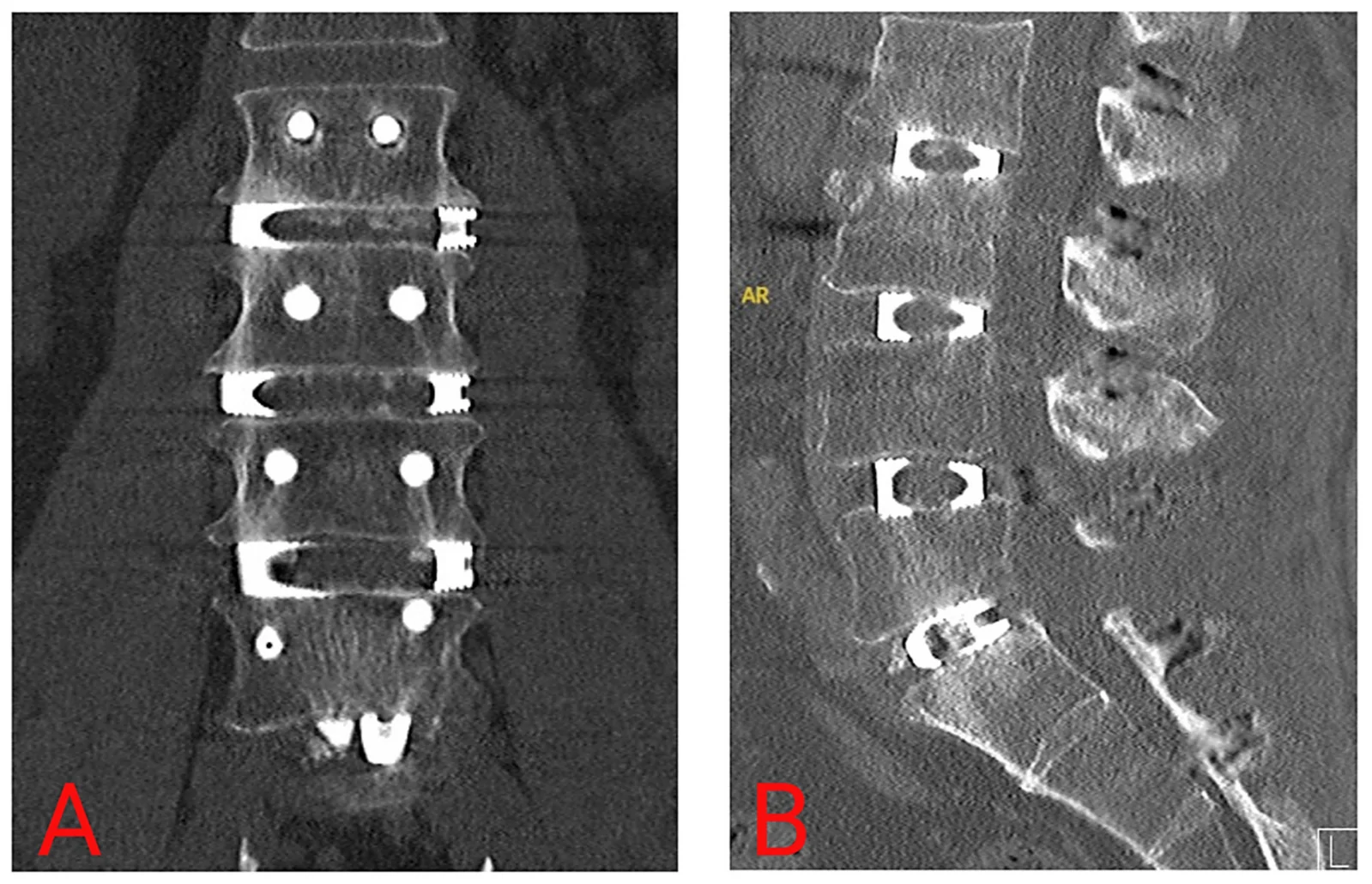
Coronal (**A**) and sagittal (**B**) computed tomography (CT) scans of the lumbar spine showcasing a multi-level posterior lumbar interbody fusion utilizing interbody fusion cages and pedicle screw instrumentation.

The success of spinal fusion procedures and implant stability critically depends on achieving robust osseointegration—the direct structural and functional connection between living bone and the implant surface (Figure 1) [34].

**Figure 2:**
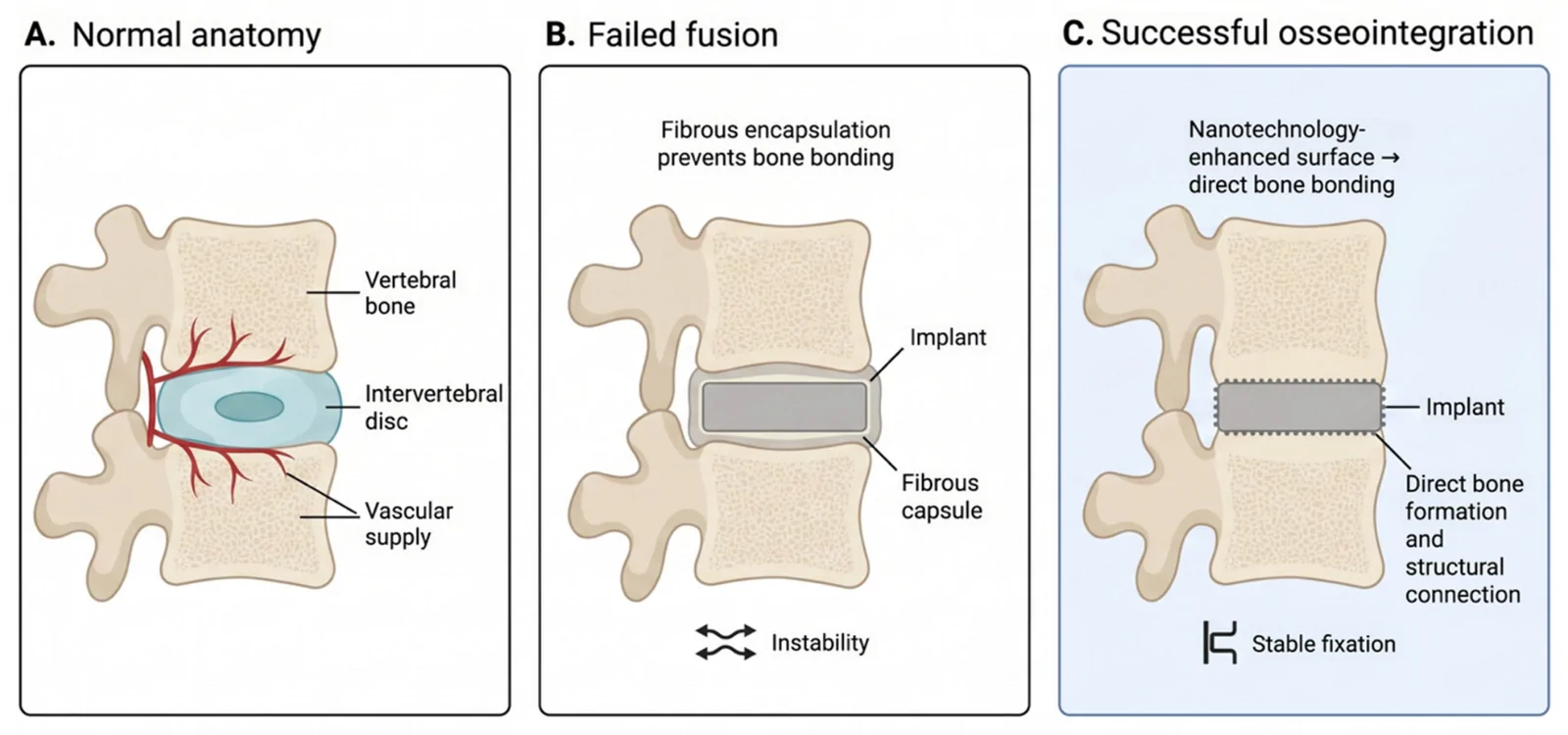
Schematic representation of the spinal fusion and osseointegration process. (**A**) Normal joint anatomy showing the intervertebral disc and adjacent vertebral bone with vascular supply. (**B**) Failed fusion characterized by the formation of a fibrous capsule at the bone-implant interface, leading to instability. (**C**) Successful osseointegration, where nanotechnology-enhanced surface properties promote direct bone formation and structural connection between the living bone and the implant. Created in BioRender. Pang, A.S.-R. (2026) https://BioRender.com/h19tfs3, accessed on 12 August 2026.

The process of osseointegration involves a complex cascade of events, beginning with protein adsorption and blood clotting at the implant surface, followed by site infiltration and biological recognition by MSCs and osteoblasts, and culminating in bone formation by these cells at the interface, thus creating an intimate bond between bone and implant (Figure 2). These events are directly and indirectly influenced by the surface properties of the device, making them key determinants of the implant’s outcome in vitro, in vivo, and clinically [11].

**Figure 3:**
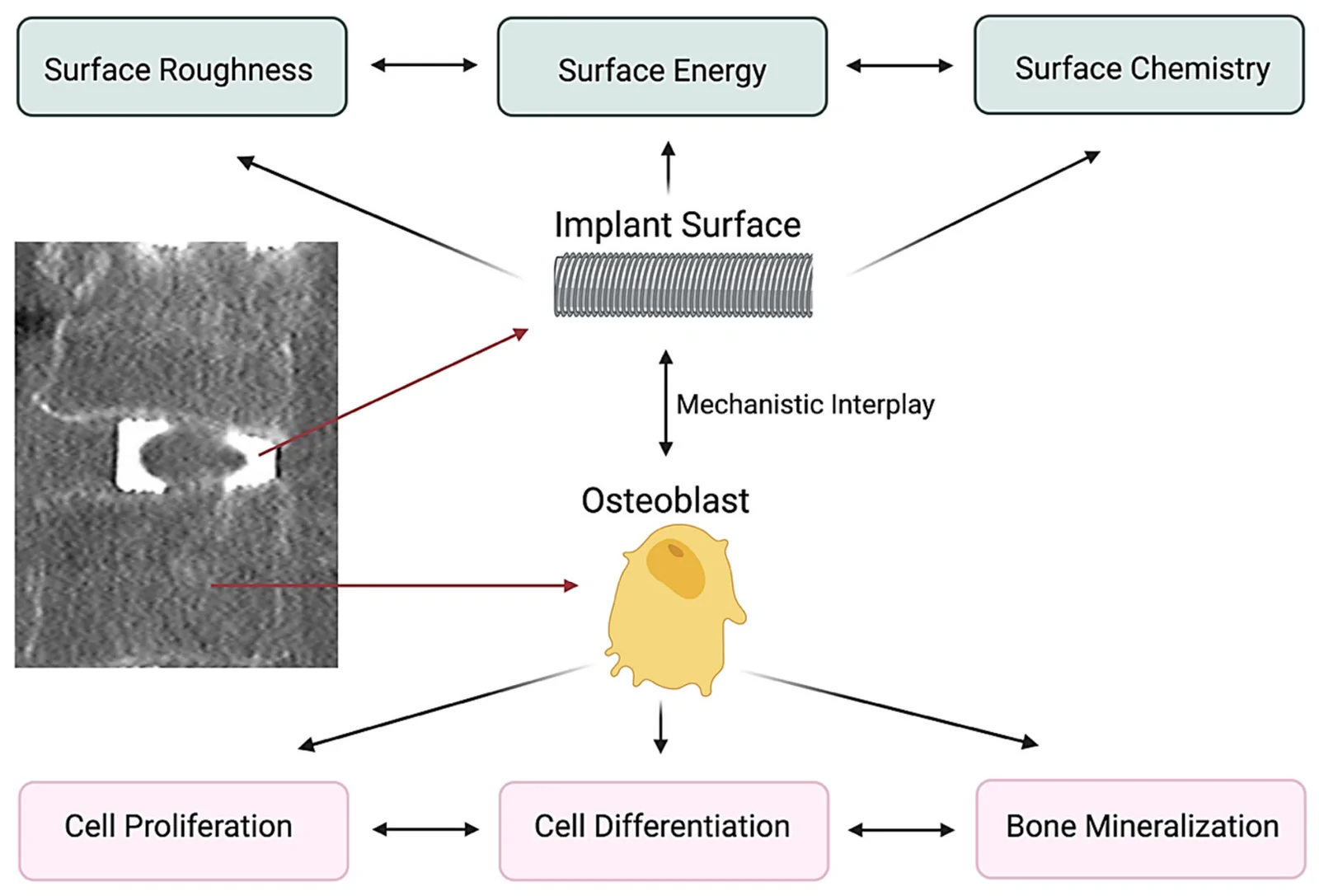
Mechanistic interplay between implant surface properties and osteoblast response. This diagram illustrates the direct and indirect interactions between specific surface parameters, including roughness, surface energy, and chemistry, and the biological cascade required for successful osseointegration. These properties govern initial protein adsorption, which subsequently directs critical osteoblast behaviours such as cellular proliferation, differentiation, and final bone mineralization. Created in BioRender. Pang, A.S.-R. (2026) https://BioRender.com/z78jeeg, accessed on 12 August 2026.

Implant surface roughness, surface energy, and surface chemistry are critical determinants of this process (Figure 3). Surface roughness and nanostructures enhance protein adsorption and blood coagulation, which are essential for early osseointegration, as demonstrated in vitro [35,36]. In vitro studies have demonstrated that increased surface energy and hydrophilicity further promote selective adsorption of osteogenic proteins such as fibronectin, modulating early cell responses and improving osteogenic outcomes [35,37]. These early surface-mediated events set the stage for subsequent cellular responses observed in vivo.

In animal models, these surface properties direct the osteoinductive pathway by modulating MSC and osteoblast behaviour, including upregulation of key transcription factors such as *Osterix*, *RUNX2*, and *BMP2*, which are essential for osteogenic differentiation and bone matrix production [38,39,40,41]. Surfaces with combined micro- and nanoscale roughness synergistically mimic the natural bone ECM, resulting in enhanced cell adhesion, proliferation, and osteogenic gene expression compared to either feature alone [11,39,42,43].

Nanostructured modifications of titanium and other metallic implant surfaces have demonstrated superior osteogenic properties compared to conventional smooth or microrough surfaces in vitro and in animal studies. Comprehensive mechanical and biological analysis of three-dimensional (3D)-printed porous interbody cages demonstrated that microporous endplates reduce block stiffness by 24.9% (*p* < 0.0001), while body lattice and microporous endplate feature together reduce cage stiffness and stress shielding risk. In an ovine interbody fusion model, these porous titanium cages supported bone ingrowth (54% to 83% in central aperture) and segmental mechanical stability as early as 12 weeks [44]. In a complementary study evaluating cage material and design effects on subsidence and osseointegration, porous titanium cages demonstrated the least subsidence displacement (0.195 mm) compared to solid polyetheretherketone (PEEK) (0.328 mm) and solid titanium (0.538 mm) cages under clinically relevant dynamic loading. Histologically, only porous titanium supported bony ingrowth with marked bone formation within interconnected pores through 12 weeks and was the only group showing significant improvement in osseointegration from 4 to 12 weeks on push-out testing. The superior subsidence resistance of microporous titanium compared to PEEK, despite reduced bulk stiffness, reflects the critical role of osseointegration in load distribution. While microporous architecture reduces titanium stiffness by 24.9%, the biological fixation achieved through bone ingrowth into microporous endplates creates a mechanically continuous interface that distributes compressive loads across a broader surface area. In contrast, PEEK cages rely solely on mechanical friction without biological integration permitting micromotion and progressive endplate remodelling under cyclic loading that drives subsidence despite lower material stiffness [45]. Titania nanotube arrays have demonstrated significant effects on osseointegration in preclinical studies. TiO_2_ nanotubes on titanium surfaces improve bone bonding 9-fold (*p* = 0.008) and increase mineralization rate approximately 3-fold compared to non-treated surfaces, while also reducing initial adhesion and colonization of Staphylococcus aureus [46]. Titania nanotube arrays with diameters ranging from 15 to 120 nm can significantly influence blood clot formation and inflammatory responses during the early healing phase, with specific nanotube dimensions (particularly 15 nm) promoting favourable osteoimmunomodulatory environments that accelerate bone regeneration [47]. Micro/nano-biomimetic coatings that replicate the hierarchical structure of natural bone ECM orchestrate multiple regenerative processes simultaneously, including osteogenesis, angiogenesis, and immunomodulation, leading to enhanced osseointegration [10,48].

The coronal and sagittal CT scans in Figure 1 demonstrate the high-load macro-environment of a multi-level posterior lumbar interbody fusion. To minimize the risk of cage subsidence into these vertebral endplates, nanostructured modifications can be engineered to reduce block stiffness and mimic natural bone architecture. The mechanisms underlying nanostructure-mediated osseointegration involve direct regulation of gene expression in adherent cells. In mice, nanoscale surface topographies were shown to upregulate key osteogenic transcription factors, particularly *Osterix*, along with downstream markers including alkaline phosphatase, bone sialoprotein, osteocalcin, and dentin matrix protein 1 [38]. This nanoscale-mediated osteoinductive pathway demonstrates that surface properties alone, independent of chemical composition, can direct stem cell fate and promote bone formation. Furthermore, the synergistic combination of microroughness and nanostructures produces superior outcomes compared to either modification alone, suggesting that hierarchical surface architectures most effectively recapitulate the natural bone microenvironment [11].

Notably, nano-HA coatings have also demonstrated efficacy in compromised bone models; in diabetic rats, nano-HA-coated implants showed higher bone-implant contact at 7 days and increased Runx2, ALP, and osteocalcin expression compared to uncoated controls [22].

Clinical studies comparing nanostructured materials to conventional implants provide encouraging but preliminary evidence. In a long-term clinical follow-up study (minimum 8 years, *n* = 107), Hu et al. (2019) demonstrated that nano-hydroxyapatite/polyamide-66 (n-HA/PA66) cages achieved similar fusion rates to titanium mesh cages (97% vs. 94%) but with significantly lower subsidence (2.4 mm vs. 3.0 mm, *p* < 0.01) and reduced rates of severe subsidence >3 mm (18.2% vs. 40.4%, *p* < 0.01), along with superior JOA functional scores [49]. A prospective clinical study by Li et al. (2025) comparing three implant types in anterior cervical corpectomy and fusion demonstrated that n-HA/PA66 cages had significantly reduced subsidence rates (20%) compared to titanium mesh cages (45%) at two-year follow-up, with functional spinal unit height loss of 2.11 mm versus 3.07 mm respectively [50]. 3D-printed vertebral bodies showed even better performance with only 10% subsidence and 1.46 mm height loss [50]. These clinical outcomes align with preclinical findings showing that nano-HA coatings increase fusion rates, new bone formation, and biomechanical properties while enhancing BMP-2 and TGF-β1 expression in the fusion region [28,51,52,53,54].

However, the translation from promising preclinical data to consistent clinical outcomes faces several obstacles. The evidence for nanostructured surface modifications improving osseointegration is compelling at the preclinical level, yet clinical validation remains limited. Long-term safety data for nanocoatings remains insufficient, particularly regarding potential nanoparticle release, accumulation in distant organs, and chronic inflammatory responses [8]. The regulatory landscape classifies medical devices containing nanomaterials as Class III (highest risk) under European Regulation 2017/745 unless the nanomaterial is encapsulated or bound such that internal exposure potential is negligible [55]. This classification reflects legitimate concerns about the unique toxicological profiles of nanomaterials compared to their bulk counterparts, driven by high surface area-to-volume ratios and enhanced reactivity [56]. A summary of key studies on structural nanotechnology modalities and their main findings is tabulated (Table 2).

### 2.3. Nano-Drug Delivery Systems for Intervertebral Disc Degeneration

IVDD represents the most common aetiology of low back pain, affecting more than 80% of adults at some stage of life and imposing substantial economic burdens on healthcare systems worldwide [9,12]. The pathophysiology of IVDD involves progressive destruction of the ECM, phenotypic changes in disc cells, loss of viable cells, increased cellular senescence and death, and excessive inflammatory responses [9]. Current treatment modalities, including non-steroidal anti-inflammatory drugs, analgesics, and surgical interventions, are primarily aimed at symptom control and functional improvement rather than restoration of native disc biology [9,12]. The complex pathogenesis of IVDD, involving progressive ECM destruction, phenotypic changes in disc cells, cellular senescence, and excessive inflammation, requires multifaceted therapeutic approaches that NDDSs are uniquely positioned to deliver.

**Figure 4:**
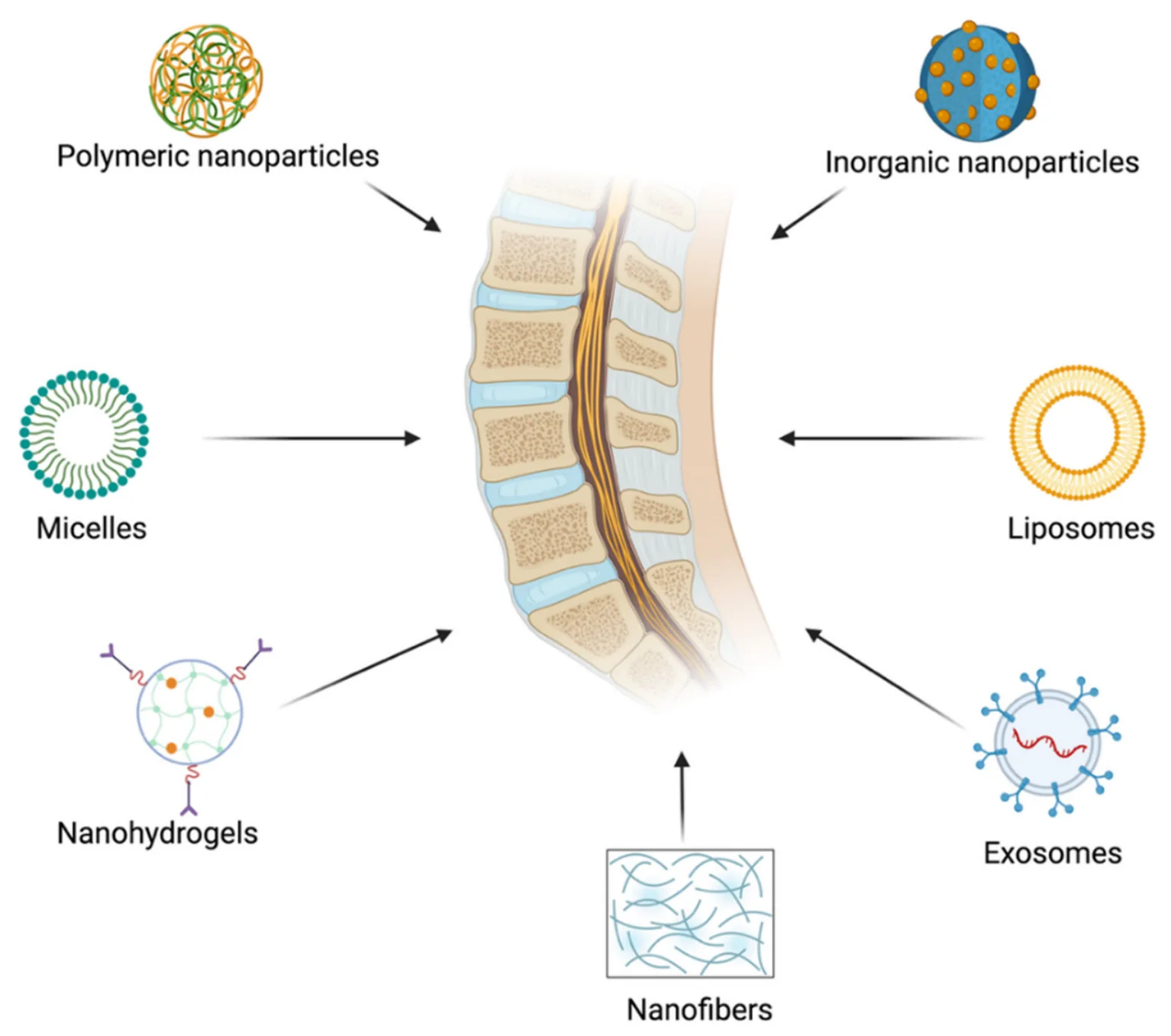
Overview of the diverse nano-drug delivery systems (NDDSs) currently developed for the treatment of intervertebral disc degeneration (IVDD). These modalities include polymeric nanoparticles, polymer micelles, nanohydrogels, inorganic nanoparticles, liposomes, exosomes, and nanofibers. Each platform is engineered to improve drug retention, cross cellular barriers, and provide sustained therapeutic release within the avascular disc environment. Created in BioRender. Pang, A.S.-R. (2026) https://BioRender.com/rg811vs, accessed on 12 August 2026.

NDDSs has potential in terms of IVDD treatment, offering several distinct advantages over conventional pharmacotherapy administered systemically or via in situ intervertebral disc injection. These systems demonstrate excellent biocompatibility and biodegradability, can cross cellular barriers to improve drug retention, provide sustained release that prolongs effective drug concentrations while reducing administration frequency. For example, PLGA microspheres with layer-by-layer assembly enabling dual release of dexamethasone and TGF-p3 achieved 76% disc height recovery and 76% MRI signal intensity at 24 weeks in a rat IVDD model [57]. These systems can be engineered from natural materials that mimic cartilage and disc-related matrices as shown in many in vitro and in vivo animal studies [9,12,58]. Macroporous biohybrid hydrogels have been developed that match native NP mechanical properties while supporting collagen II and glycosaminoglycan deposition by human NP cells in vitro [59]. Multiple nanocarrier platforms (Figure 4) have been developed for IVDD applications, including liposomes, polymeric nanoparticles, inorganic nanoparticles, polymer micelles, nanofibers, nanohydrogels, and exosomes, each offering unique properties for specific therapeutic applications [12,60]. These systems, although in pre-clinical stages, offer several potential advantages: sustained drug release that prolongs effective concentrations, ability to cross cellular barriers and activate specific transport mechanisms, biodegradability with excellent biocompatibility, and capacity to respond to pathological microenvironmental cues such as elevated reactive oxygen species and acidic pH [9,61,62].

Recent advances in NDDS design have focused on creating stimuli-responsive systems that respond to the pathological microenvironment of degenerated discs [61,63]. For example, in mice, hydrogel-based drug delivery systems incorporating celecoxib-loaded polydopamine nanoparticles can respond to elevated reactive oxygen species and acidic pH characteristic of degenerated discs, enabling on-demand and sustained drug release [64]. Similarly, polyphenol nanosphere-hydrogel composites delivering antagomir-21 have demonstrated ECM regeneration and ROS scavenging capabilities in rat IVDD models [61]. These systems effectively reduce inflammation and oxidative stress in nucleus pulposus cells by modulating the COX2-PGE2-NFκB and TNFα-P38-MAPK signalling pathways, ultimately restoring disc structure and function [64]. The convergence of stimuli-responsive release and multi-pathway targeting exemplified by these systems addresses the multifactorial pathogenesis of IVDD more comprehensively than single-agent therapies, representing a paradigm shift from symptomatic to regenerative treatment. Intelligent nanoliposomes have also been developed to inhibit neurovascular ingrowth and alleviate discogenic pain via the Hippo-YAP pathway [65]. In a rat IVDD model, nanohybrid peptide hydrogels incorporating two-dimensional nanomaterials with enzyme-like functions were shown to simultaneously scavenge pro-inflammatory reactive oxygen species, promote ECM remodelling, and deliver pro-regenerative cytokines in a sustained manner [66].

Combination strategies integrating nanostructured scaffolds with stem cell delivery have also shown promise. A chitosan hydrogel incorporating PLGA microspheres for sustained GDF5 release combined with nucleus pulposus-derived stem cells (NPSCs) achieved effective NP regeneration in rats, outperforming bone marrow-derived MSCs [67]. Exosome and extracellular vesicle (EV)-based therapies represent an emerging class of biological NDDSs for IVDD. MSC-derived exosomes have been shown to suppress NLRP3 inflammasome activation and restore mitochondrial function in rabbit models [68]. Hypoxia-preconditioned EVs delivering miR-17-5p promoted NPC proliferation via the TLR4/PI3K/AKT pathway [69], Hypoxia-preconditioned exosome-hydrogel composites have also demonstrated ECM regeneration through the PPARγ-autophagy axis, achieving structural and functional IVD repair in rat models [70]. Engineered EVs carrying FOXF1 restored disc height and hydration with sex-specific pain reduction [71]. A 2026 meta-analysis, conducted by Wei et al., of 19 preclinical studies (n = 305) confirmed that SC-derived EVs improved disc height index by 12.8% and Pfirrmann grade by 1.5 points, with hydrogel-based delivery reducing heterogeneity [72].

Nanostructured scaffolds and hydrogels have also been developed as cell and drug delivery platforms for IVDD. Nanostructured gelatin colloidal gels improved disc height, GAG content, and MRI index while preventing MSC leakage in rabbit models [73]. Self-assembling peptide nanofiber hydrogels upregulated collagen II, aggrecan, and SOX-9 in vitro [74,75], while nanofiber-reinforced alginate hydrogels provided leak-proof PRP delivery with reduced degeneration at 8 weeks [76]. Electrospun nanofibrous microscaffolds and laser-structured PLGA microscaffolds enhanced cell viability and retention for injectable disc repair [77,78].

Despite these advances, translation of NDDSs to clinical IVDD treatment faces substantial hurdles. The avascular nature of the intervertebral disc creates challenges for systemic delivery, necessitating direct injection approaches that raise questions about optimal dosing, injection frequency, and long-term retention. The short half-life of bioactive substances limits therapeutic duration, although NDDSs partially address this through controlled release mechanisms [9]. Furthermore, identifying specific molecular targets for drug and gene therapy remains challenging given the multifactorial nature of IVDD pathogenesis [9,79]. A summary of key studies on nanotechnology-based therapeutics for intervertebral disc degeneration and their main findings is tabulated (Table 3).

## 3. Biocompatibility and Safety Considerations

While properly designed nanomaterials demonstrate excellent biocompatibility in spinal applications, the long-term health effects of nanomaterials remain poorly understood, necessitating extensive research regarding clinical safety [7,80,81]. Nanotechnology offers tremendous promise for spinal surgery applications, though careful consideration of biocompatibility and safety remains paramount. Nanomaterials can exhibit different toxicological profiles compared to their bulk counterparts due to their high surface area-to-volume ratios, enhanced reactivity, and ability to cross biological barriers. The biocompatibility-neurotoxicity relationship of nanoparticles depends on multiple factors, including particle size, shape, surface chemistry, degradability, and dose [7,56]. Concerns about potential carcinogenicity, particularly for fibrous nanomaterials with size and shape similarities to asbestos, require careful evaluation [82].

Graphene oxide-based nanocomposites functionalized with chitosan and polyethylene glycol show non-toxic profiles and actually enhance MSC proliferation by approximately 10%, while promoting functional recovery in spinal cord injury models [83]. Nanostructured polymers, metals, ceramics, and carbon materials have also been explored in orthopaedic and spinal applications, with several studies reporting encouraging biocompatibility and safety profiles [13].

The development of biodegradable nanomaterials that undergo controlled degradation into biocompatible byproducts represents one strategy for mitigating long-term safety concerns. Stimuli-responsive nanomaterials that activate only in response to specific pathological conditions may reduce off-target effects and systemic exposure [62,84,85]. Nevertheless, comprehensive long-term clinical studies are necessary to establish the safety profile of nanocoatings and nanoparticle-based therapies, particularly regarding potential accumulation, biodistribution to distant organs, and chronic inflammatory responses, before widespread clinical adoption can be recommended [8].

## 4. Regulatory and Manufacturing Challenges: The Translation Gap

Recent advances in lipid nanoparticle-based therapeutics, such as mRNA vaccines, have helped inform regulatory considerations for nanoscale delivery systems [56]. Translation of nanotechnology from preclinical studies to clinical applications requires manufacturing under GMP conditions, standardization of characterization methods, and comprehensive assessment of long-term outcomes [15]. Regulatory authorities including China’s National Medical Products Administration and the European Union have issued guidance documents specifying requirements for physicochemical characterization, biological evaluation, and safety assessment of nanomaterial-containing devices [86].

Standardization of characterization methods remains a critical challenge. Nanomaterials require evaluation of parameters including size distribution, shape, surface chemistry, surface area, aggregation state, degradability, and release kinetics—measurements that often lack standardized protocols across laboratories and regulatory jurisdictions [33,86]. The development of validated in vitro testing methods to advance regulatory science and reduce reliance on animal testing represents an important area of ongoing work [86].

Manufacturing challenges compound regulatory hurdles. Achieving consistent nanostructure formation across large-scale production batches, maintaining sterility during nanomaterial incorporation, and ensuring long-term stability of nanocoatings under physiological conditions all require sophisticated quality control systems. Production and development costs represent another barrier to commercialization, as nanomaterial synthesis, characterization, and regulatory compliance substantially increase production expenses compared to conventional materials [33,87].

### A Coordinated Roadmap to Accelerate Clinical Translation

The decade-long stagnation in nanotechnology commercialisation for spinal applications reflects systemic barriers rather than scientific limitations. Overcoming this translational impasse requires coordinated action across five strategic priorities that address regulatory, manufacturing, and evidence-generation challenges simultaneously.

Establishing global clinical registries for long-term safety surveillance is of paramount importance. National and international implant registries have proven instrumental in postmarket surveillance for orthopaedic surgery and cardiac devices, enabling early detection of safety signals, real-world effectiveness assessment, and regulatory decision-making. A dedicated global registry for nanostructured spinal implants and NDDS should capture standardised elements including patient demographics, device characteristics (nanostructure specifications, coating composition, etc.) surgical details, and longitudinal outcomes (fusion rates, subsidence, adverse events, revision procedures) [88,89]. Registry infrastructure must ensure sustainable funding, linkage to electronical health records for real-time data capture, public data availability, and incentives for clinician participation. The FDA’s National Evaluation System for health Technology (NEST) and the International Consortium of Orthopaedic Registries provide proven models for integrating diverse data sources while maintaining patient privacy [88,90]. Multicentre translational consortia focused on nanotechnology in spinal surgery should be formed to pool resources and expertise. International harmonisation of nanomaterial characterisation standards safety assessment protocols, and minimal dataset requirements would reduce redundant testing and accelerate global market access.

Implementation of this roadmap requires sustained commitment from funding agencies, academic researchers, clinical investigators, industry partners, and regulatory scientists to design pragmatic trials, share manufacturing protocols, and standardise characterisation methods. Only through such coordinated efforts can the field bridge the gap between promising preclinical findings and routine clinical application.

## 5. Future Directions: Smart Nanomaterials and Personalized Medicine

One potential direction for nanotechnology in spinal applications lies in the development of increasingly sophisticated, multifunctional systems that integrate diagnostic and therapeutic capabilities. Smart biomaterials with stimuli-responsive behavior represent a particularly promising direction, including materials that respond to pH, temperature, mechanical stress, or specific molecular signals characteristic of pathological conditions [33]. These materials could provide on-demand drug release, potentially enabling monitoring of local biological responses, and adaptive mechanical properties that evolve with tissue regeneration.

The integration of 3D bioprinting offers routes to patient-specific grafts with predictable performance. Artificial-intelligence guided algorithms can optimize nanoparticle design parameters, including size, shape, surface chemistry, and degradation kinetics, based on desired biological outcomes, accelerating the development cycle from concept to clinical application [15,85]. In parallel, 3D printing enables the fabrication of complex geometries, while nanotechnology can introduce hierarchical porosity and spatially controlled nanoparticle distribution, creating scaffolds that recapitulate the natural bone microenvironment while delivering therapeutic agents in precise spatiotemporal patterns [8].

Multimodal nanoparticles that integrate different nanoparticle types and combine surface roughness, ion release, and chemical cues offer synergistic effects by targeting both intracellular processes and the bone microenvironment [29]. These dual-function approaches modulate inflammation, oxidative stress, and cellular signalling simultaneously, potentially achieving superior outcomes compared to single-mechanism interventions. The development of self-reporting nanomaterials that provide real-time feedback on healing status through optical, magnetic, or other signals could, in principle, provide feedback on the local biological environment and enable more personalized adjustment of treatment strategies [15].

## 6. Limitations and Knowledge Gaps

Several important limitations constrain current understanding of nanotechnology applications in spinal surgery. Most evidence derives from in vitro studies and animal models, with limited clinical data available to validate preclinical findings in human patients [7]. The translation from animal models to human clinical outcomes remains uncertain, particularly given differences in biomechanical loading, healing kinetics, and immune responses between species. In addition, long-term data are scarce, leaving unanswered questions regarding the durability of nanostructured coatings, potential late complications, and sustained therapeutic efficacy [8].

### 6.1. Application-Specific Limitations: Structural vs. Therapeutic Nanotechnology

While both structural and therapeutic nanotechnology approaches face common challenges in regulatory approval and manufacturing standardisation, each confronts distinct translational barriers that reflect fundamental differences in their clinical applications and biological environments.

Nanostructured implant surfaces must withstand the demanding biomechanical environment of the spine, where cyclic loading, shear forces, and micromotion create risks of coating delamination, wear particle generation, and loss of nanostructural integrity over time. The optimal design parameters for nanostructured surfaces, including ideal nanotube diameter, spacing, depth, and combination with microroughness, remain incompletely defined and likely vary depending on specific clinical applications and patient populations [11].

The heterogeneity of preclinical fusion models further complicates comparative assessment of nanomaterial efficacy. Studies employ different species (rat vs. rabbit), fusion techniques (posterolateral vs. interbody), observation periods, and control groups (autograft, scaffold alone, etc.), making direct cross-study comparisons challenging. Standardized preclinical models with consensus outcome measures are needed to definitively establish which nanomaterial properties optimize fusion outcomes.

NDDSs face fundamentally different challenges rooted in the unique biology of IVD tissue. The avascular nature of the disc creates barriers to systemic delivery and limits nutrient supply for sustained cellular responses, necessitating direct intradiscal injection approaches that raise questions about optimal dosing, injection frequency, and long-term retention within the disc space. For NDDSs, questions persist regarding optimal nanocarrier selection for specific therapeutic agents, ideal particle size for disc penetration, and strategies for achieving targeted delivery to degenerated discs while minimizing systemic exposure [9]. Furthermore, the interaction between nanomaterials and the complex immune microenvironment of the spine requires further investigation, particularly regarding macrophage polarization and the foreign body response [15]. Beyond these biological considerations, challenges related to large-scale manufacturing, regulatory approval, cost of production, and long-term safety evaluation may further limit the near-term clinical adoption of nanotechnology-based spinal therapies.

### 6.2. Spinal Cord Injury: Unique Translational Barriers

Nanotechnology applications for spinal cord injury face distinct challenges that differ fundamentally from bone regeneration and disc degeneration. The blood-spinal cord barrier presents a formidable obstacle to systemic nanoparticle deliver, requiring either direct intraspinal injections or barrier-disrupting strategies that carry inherent risks of secondary injury. The injury microenvironment itself is extraordinarily complex, characterized by dynamic phases of acute inflammation, glial scar formation, cystic cavity development, and chronic neuroinflammation—each requiring different therapeutic interventions at specific temporal windows. Most spinal cord injury nanotechnology research remains in early preclinical stages, with studies conducted predominantly in rodent contusion or transection models that incompletely recapitulate human spinal cord injury heterogeneities [13]. Critical knowledge gaps include optimal nanoparticle size for penetrating glial scars, strategies for achieving sustained therapeutic concentrations across injury epicenters, and methods for promoting axonal regeneration across cystic cavities. These complexities explain why spinal cord injury nanotheranostics lag behind applications in spinal fusion and disc regeneration.

## 7. Conclusions

Nanotechnology has the potential to enhance several aspects of spinal surgery through enhanced osseointegration, targeted drug delivery, improved fusion outcomes, and regenerative medicine applications. The mechanistic understanding of how nanoscale features influence cellular behavior has advanced substantially, revealing that surface topography, controlled ion release, and modulation of inflammatory responses can be precisely engineered to promote healing. Early clinical studies suggest potential benefits supporting the efficacy of nanostructured materials in reducing implant subsidence and enhancing fusion rates compared to conventional devices.

However, significant barriers must be overcome before nanotechnology can fulfil its promise in routine clinical practice. Regulatory evaluation of nanomaterials continues to evolve, manufacturing processes require greater standardization to ensure reproducibility and safety, and robust long-term clinical studies are needed to establish durability and identify potential late complications. In addition, the cost-effectiveness of nanotechnology-based interventions compared to conventional treatments requires rigorous health economic evaluation.

The path forward requires collaborative efforts among materials scientists, clinicians, regulatory agencies, and industry partners to translate promising experimental findings into safe, effective, and accessible clinical therapies. By addressing current knowledge gaps through well-designed clinical trials, standardized characterization methods, and scalable manufacturing strategies will be essential to support the responsible integration of nanotechnology into spinal care. The ultimate goal of realizing the clinical potential of nanoscale interventions in spinal surgery may seem distant but achievable and will require continued progress in translational research and careful clinical validation.

## Figures and Tables

**Table 1:** Comparative outcomes of nanoparticle-enhanced spinal fusion in preclinical models.

Authors	Year	Species	Model	Nanomaterial	Control	Timeline	Fusion Rate (Nano)	Fusion Rate (Control)	Reference(s)
Notani	2018	Rat	PLF	Carbon nanofibers + BMP-2	BMP-2 alone	8 wks	45.5%	0.0%	[25]
Conway	2021	Rabbit	PLF	Silicated CaP putty	Autograft	12–26 wks	87.5% (12 wks), 100% (26 wks)	100% (26 wks)	[27]
Zhuo	2018	Rabbit	PLF	Nano-HA/BCP + CMF	BCP alone	Not specified	Increased vs. control	Baseline	[28]

Abbreviations: BCP, biphasic calcium phosphate; BMP-2, bone morphogenetic protein-2; CaP, calcium phosphate; CMF, combined magnetic field; HA, hydroxyapatite; PLF, posterolateral fusion; wks, weeks.

**Table 2:** Structural nanotechnology modalities with potential application in spinal surgery, their clinical advantages, and challenges for clinical translation.

Authors	Year	Study Type	Model	Key Findings	Reference(s)
de Oliveira et al.	2021	Preclinical	Diabetic rat tibia	Nano-HA coating: higher BIC at 7 days; increased Runx2, ALP, osteocalcin expression	[22]
Conway et al.	2021	Preclinical	Rabbit PLF	Nanosynthetic silicated CaP: 87.5% fusion at 12 weeks; 100% fusion at 26 weeks; equivalent to autograft	[27]
Zhuo et al.	2018	Preclinical	Rabbit PLF	Nano-HA/BCP + CMF: increased fusion rate; enhanced BMP-2 and TGF-β1 expression	[28]
Toop et al.	2023	RCT (n = 50)	Human TLIF	Nano-etched Ti cages: 84% BSF-3 fusion at 6 months vs. 20.6% PEEK (*p* < 0.001); subsidence 20.8% vs. 41.4%	[30]
Shirazi et al.	2024	Preclinical + scRNA-seq	Rat model	Nanoscale topography: higher BIC at 21 days; earlier RUNX2/BMP2 expression; reduced inflammation	[39]
Xu et al.	2016	Preclinical	In vivo	Micro/nano-textured SLM Ti: BIC nearly double native-SLM at 8 weeks; ECM-like nanonet most effective	[43]
Fogel et al.	2022	Preclinical + mechanical	Ovine interbody	3D-printed porous Ti: 100% rigid fusion at 26 weeks; bone ingrowth into microporous endplates	[44]
Fogel et al.	2022	Preclinical + mechanical	Ovine model	Porous Ti: least subsidence (0.195 mm) vs. PEEK (0.328 mm) vs. solid Ti (0.538 mm)	[45]
Su et al.	2018	Preclinical review	Rabbit model	TiO_2_ nanotubes: 9-fold improvement in bone bonding (*p* = 0.008); 3× mineralization rate	[46]
Bai et al.	2018	Preclinical	In vivo	HA nanoparticles vs. nano-rods: nanoparticles enhanced osteo-/angio-genesis and osteoimmunomodulation	[48]
Hu et al.	2019	Retrospective (n = 107, 8-year)	Human ACCF	n-HA/PA66: subsidence 18.2% vs. 40.4% TMC (*p* < 0.01); 97% fusion rate	[49]
Li et al.	2025	Prospective (n = 60)	Human ACCF	n-HA/PA66 cage: subsidence 10–20% vs. 45% TMC (*p* = 0.031); FSU height loss 1.46 mm vs. 3.07 mm	[50]
Almeida et al.	2023	Preclinical	Sheep model (low-density bone)	Nano-HA coating: superior BIC and BAFo at 28 days vs. dual acid-etched surface (*p* < 0.01)	[51]
Bigi et al.	2008	Preclinical	Rabbit cortical bone	Nanocrystalline HA coating: highest osteointegration at 4 and 12 weeks; affinity index 37% vs. 26%	[52]
Lu et al.	2020	Preclinical	In vivo	Electrodeposited nano-HA: enhanced early osseointegration; greater bonding strength vs. plasma-sprayed HA	[53]
Yamada et al.	2012	Preclinical	Rat model	Nanopolymorphic crystalline HA: increased BIC and bone volume; reduced soft tissue intervention	[54]

Abbreviations: ACCF, anterior cervical corpectomy and fusion; ALP, alkaline phosphatase; BAFo, bone area fraction occupancy; BCP, biphasic calcium phosphate; BIC, bone-to-implant contact; BMP-2, bone morphogenetic protein 2; BSF-3, Bridwell-Lenke spinal fusion grade 3; CaP, calcium phosphate; CMF, combined magnetic field; ECM, extracellular matrix; FSU, functional spinal unit; HA, hydroxyapatite; n-HA/PA66, nano-hydroxyapatite/polyamide-66; Nano-HA, nano-hydroxyapatite; PEEK, polyetheretherketone; PLF, posterolateral lumbar fusion; RCT, randomized controlled trial; RUNX2, runt-related transcription factor 2; scRNA-seq, single-cell RNA sequencing; SLM, selective laser melting; TGF-β1, transforming growth factor beta 1; Ti, titanium; TiO_2_, titanium dioxide; TLIF, transforaminal lumbar interbody fusion; TMC, titanium mesh cage.

**Table 3:** Nanotechnology therapeutic for intervertebral disc degeneration.

Authors	Year	Study Type	Model	Nanocarrier Type	Key Findings	Ref.(s)
Liang et al.	2013	Preclinical	Rat IVDD	PLGA microspheres	Dual release dexamethasone + TGF-β3: 76% disc height recovery; 76% MRI signal at 24 weeks	[57]
Zhang et al.	2018	Preclinical	Rat IVDD	Albumin/heparin nanoparticles	SDF-1α delivery: induced MSC migration; better AF and NP regeneration	[58]
Brissenden et al.	2023	In vitro	Human NP cells	Macroporous biohybrid hydrogel	Matched NP mechanical properties; supported collagen II and GAG deposition	[59]
Teixeira et al.	2016	Ex vivo	Bovine IVDD	Chitosan/poly-γ-glutamic acid NPs	Diclofenac delivery: downregulated IL-6, IL-8, MMP1, MMP3; promoted ECM synthesis	[60]
Wang et al.	2023	Preclinical	Rat IVDD	Polyphenol nanospheres + hydrogel	Antagomir-21 delivery: ECM regeneration; ROS scavenging; downregulated TNF-α	[61]
Yang et al.	2023	Preclinical	Rat nucleotomy	3D porous hybrid protein nanoscaffold	Suppressed inflammation; restored ECM; long-term pain reduction	[63]
Li et al.	2026	Preclinical	Murine IVDD	Intelligent nanoliposome	Inhibited neurovascular ingrowth; alleviated pain via Hippo-YAP pathway	[65]
Conley et al.	2023	Preclinical	Rat nucleotomy	Nanohybrid peptide hydrogel (NHPH)	ROS scavenging; ECM remodelling; NP cell differentiation; pain reduction	[66]
Ma et al.	2024	Preclinical	Rat IVDD	DNPM/chitosan hydrogel + PLGA microspheres	GDF5 + NPSCs: effective NP regeneration; superior to BMSCs	[67]
Xia et al.	2019	Preclinical	Rabbit IVDD	MSC-derived exosomes	Suppressed NLRP3 inflammasome; restored mitochondria; prevented degeneration	[68]
Zhou et al.	2022	Preclinical	Rat IVDD	Hypoxic MSC-derived sEVs	miR-17-5p delivery: promoted NPC proliferation via TLR4/PI3K/AKT pathway	[69]
Sun et al.	2025	Preclinical	Rat IVDD	Hypoxia-preconditioned exosome-hydrogel	ECM regeneration via PPARγ-autophagy axis; structural-functional IVD repair	[70]
Tang et al.	2024	Preclinical	In vivo DBP model	Engineered extracellular vesicles	FOXF1 gene delivery: sex-specific pain reduction; restored disc height and hydration	[71]
Wang et al.	2021	Preclinical	Rabbit IVDD	Nanostructured gelatin colloidal gels	MSC delivery: improved disc height, GAG content, MRI index; prevented cell leakage	[73]
Han et al.	2022	In vitro	hNPMSCs	Self-assembling peptide nanofiber hydrogel	Sa12b-modified: upregulated collagen II, aggrecan, SOX-9; inhibited ASICs	[74]
Wan et al.	2016	In vitro	Human NP cells	Self-assembling peptide hydrogel	Restored NP phenotype; increased aggrecan and collagen II deposition	[75]
Li et al.	2023	Preclinical	Rat IVDD	Nanofiber-reinforced alginate hydrogel	PRP delivery: leak-proof; reduced radiographic/MRI degeneration at 8 weeks	[76]
Nakielski et al.	2023	Preclinical	Pig model	Electrospun nanofibrous microscaffolds	Chitosan/CS-modified: prevented cell leakage; promoted IVD regeneration	[77]
Nakielski et al.	2025	Preclinical	Ex vivo NP model	Laser-structured PLGA microscaffolds	Enhanced cell viability and retention; injectable through small-gauge needles	[78]

Abbreviations: AF, annulus fibrosus; ASICs, acid-sensing ion channels; BMSCs, bone marrow-derived mesenchymal stem cells; CS, chondroitin sulfate; DBP, discogenic back pain; DNPM, deferoxamine-loaded nanoscale polydopamine microspheres; ECM, extracellular matrix; FOXF1, forkhead box F1; GAG, glycosaminoglycan; GDF5, growth differentiation factor 5; hNPMSCs, human nucleus pulposus-derived mesenchymal stem cells; IL-6, interleukin-6; IL-8, interleukin-8; IVD, intervertebral disc; IVDD, intervertebral disc degeneration; miR, microRNA; MMP1, matrix metalloproteinase 1; MMP3, matrix metalloproteinase 3; MRI, magnetic resonance imaging; MSC, mesenchymal stem cell; NHPH, nanohybrid peptide hydrogel; NLRP3, NOD-like receptor protein 3; NP, nucleus pulposus; NPC, nucleus pulposus cell; NPSCs, nucleus pulposus-derived stem cells; NPs, nanoparticles; PI3K/AKT, phosphoinositide 3-kinase/protein kinase B; PLGA, poly(lactic-co-glycolic acid); PPARγ, peroxisome proliferator-activated receptor gamma; PRP, platelet-rich plasma; ROS, reactive oxygen species; SDF-1α, stromal cell-derived factor 1 alpha; sEVs, small extracellular vesicles; SOX-9, SRY-box transcription factor 9; TGF-β3, transforming growth factor beta 3; TLR4, toll-like receptor 4; TNF-α, tumor necrosis factor alpha; YAP, yes-associated protein.

## Data Availability

No new data were created or analyzed in this study. Data sharing is not applicable to this article.
